# Design Expert Supported Mathematical Optimization and Predictability Study of Buccoadhesive Pharmaceutical Wafers of Loratadine

**DOI:** 10.1155/2013/197398

**Published:** 2013-05-28

**Authors:** Prithviraj Chakraborty, Surajit Dey, Versha Parcha, Shiv Sankar Bhattacharya, Amitava Ghosh

**Affiliations:** ^1^Bengal College of Pharmaceutical Sciences and Research, West Bengal University of Technology, B.R.B. Sarani, Durgapur, West Bengal 713212, India; ^2^College of Pharmacy, Roseman University of Health Sciences, 11 Sunset Way, Henderson, NV 89052, USA; ^3^S. Bhagwan Singh PG Institute of Biomedical Sciences and Research, Balawala, Dehradun, Uttarakhand 248001, India; ^4^School of Pharmaceutical Sciences, IFTM University, Lodhipur Rajput, Delhi Road (NH-24), Moradabad, Uttar Pradesh 244 102, India

## Abstract

*Objective*. The objective of this work encompasses the application of the response surface approach in the development of buccoadhesive pharmaceutical wafers of Loratadine (LOR). *Methods*. Experiments were performed according to a 3^2^ factorial design to evaluate the effects of buccoadhesive polymer, sodium alginate (*A*), and lactose monohydrate as ingredient, of hydrophilic matrix former (*B*) on the bioadhesive force, disintegration time, percent (%) swelling index, and time taken for 70% drug release (*t*_70%_). The effect of the two independent variables on the response variables was studied by response surface plots and contour plots generated by the Design-Expert software. The desirability function was used to optimize the response variables. *Results*. The compatibility between LOR and the wafer excipients was confirmed by differential scanning calorimetry, FTIR spectroscopy, and X-ray diffraction (XRD) analysis. Bioadhesion force, measured with TAXT2i texture analyzer, showed that the wafers had a good bioadhesive property which could be advantageous for retaining the drug into the buccal cavity. *Conclusion*. The observed responses taken were in agreement with the experimental values, and Loratadine wafers were produced with less experimental trials, and a patient compliant product was achieved with the concept of formulation by design.

## 1. Introduction 

In the recent times, there has been tremendous advances in drug delivery to develop newer dosage forms to improve efficacy of drugs that are currently in the market. However, regardless of the tremendous advances in alternate routes of drug delivery, oral route remains the most favoured route of administration of therapeutic agents in respect to low cost, ease of administration, and high level of patient compliance. Pharmaceutical oral wafers are an attractive route of administration because they dissolve or deaggregate spontaneously in the oral cavity, resulting in a solution or suspension without water. Effectively, it is a solid dosage form providing the convenience of a liquid dosage form. Majority of the drugs prescribed to patients are conventional tablets and capsules, and less attention has been paid to patients experiencing difficulty in swallowing (dysphagia) [1]. The pharmaceutical wafers hold potential advantages like rapid disintegration, no swallowing or chewing, no coadministration of water, accurate dosing compared to liquid products, great safety, and efficacy along with patient compliance.

Buccal mucosa, an attractive route for systemic delivery of drugs, is relatively permeable with a rich blood supply [2, 3]. A drug can be easily applied and localized at the application site and can also be eliminated from there, whenever in need [4]. Buccal delivery for the transmucosal absorption of drugs into the systemic circulation offers a number of advantages over oral delivery, especially for those drugs that have low oral bioavailability and/or those drugs that suffer from extensive first-pass metabolism in the liver.

A second-generation tricyclic H1 antihistaminic, Loratadine (LOR), is marketed for its nonsedating properties. The role of these classes of drug is to prevent and/or suppress the action of histamine, mediated by allergen in the nose and conjunctivae, thereby eliminating symptoms, that is, itching, congestion, rhinorrhoea, tearing, and sneezing [5]. Earlier reports had revealed that formulation of Loratadine as a medicated chewing gum results in an almost threefold increase in relative bioavailability. This is due to the fact that approximately 40% of the absorbed Loratadine was absorbed via the oral mucosa and thereby bypassing first-pass metabolism [6]. Hence, it is an attempt to prepare pharmaceutical wafers of Loratadine with an aim to lessen the lag time and enhance the onset of action.

In the development of a dosage form, a crucial issue is to design an optimized pharmaceutical formulation in a short time period with marginal trials. Due to the complication in the development of pharmaceutical formulations, some computer-based optimization techniques based on response surface methodology (RSM) representing the use of appropriate experimental designs and applying polynomial equation have been widely used [7, 8]. 

The aim of RSM is to discover the optimum operating conditions for a given system or the way in which a particular response is modified by a set of variables over some specific regions of interest.

Factorial designs, dealing with factors in all possible combinations, are considered to be the most efficient in estimating the influence of individual variables and their interactions using nominal experiments [9]. The applicability of factorial design in the development of pharmaceutical formulation has helped in understanding the link between the independent variables and the responses to them [10]. The independent variables are manageable, whereas responses are dependent. This supports the process of optimization by rendering an empirical model equation for the response as a function of the different variables. The technique needs minimum experimentation and time, thus establishing far more cost-effective formulation than the conventional methods of formulating dosage form.

The current study aims at developing and optimizing a fast-dissolving pharmaceutical wafer containing Loratadine, utilizing a computer aided optimization technique. Factorial 2 factor interaction model was employed to investigate the effect of sodium alginate (sodium salt of alginic acid) as a bioadhesive polymer and lactose monohydrate as an ingredient (filler) of hydrophilic matrix base [11] for formulating the wafer which will also impart a pleasant mouth fill. This is due to the fact that the two important variables, that is, bioadhesiveness imparted by the incorporation of a bioadhesive polymer and texture of wafer dosage form due to the filler, shall contribute effect on the nature and performance of the bioadhesive pharmaceutical wafers.

## 2. Experimental

### 2.1. Materials and Method

Loratadine (LOR), hydroxy propyl cellulose (HPC) (Klucel), and saccharine sodium were procured from Yarrow Chem Mumbai, India; sodium alginate, lactose monohydrate, polyethylene glycol 400 were obtained from Merck, India; sorbitol (Liquid 70%) was procured from CDH, India; glycerol was obtained from Loba Chemie, Mumbai, India. All the other chemicals and solvents used were of AR grade.

### 2.2. Experimental Design

A 3^2^ factorial design was employed where the amount of two carriers (factors) were varied at three levels as hypothesized by the design. 

The amount of sodium alginate as a bioadhesive polymer (*A*) and lactose monohydrate (*B*) were selected as factors and studied at three levels [12].  Table 1 summarizes the nine experimental runs studied, their factor combinations, and the translation of the coded level to the experimental units employed during the study. Bioadhesive force (*Y*_1_), % swelling index (*Y*_2_), disintegration time (*Y*_3_), and time taken for the release of 70% of drug (*t*_70%_ or *Y*_4_) were taken as the response variables.

### 2.3. Preparation of Bioadhesive Pharmaceutical Wafers of Loratadine

The solvent casting method is undoubtedly the most widely used manufacturing process for making orodispersible/quick dissolving thin films or wafers as depicted in the literature [13]. For the preparation of pharmaceutical wafers using solvent casting method, the base film forming polymer (2% w/v of HPC) was mixed with the required amount of sodium alginate as per the experimental design and kept for overnight soaking in distilled water containing a constant proportion of propylene glycol, glycerine, and sorbitol as plasticizers. A calculated amount of LOR dissolved in aliquot of ethanol was added in the vortex of the vigorously stirred suspension of the plasticized aqueous polymeric gel. Lactose monohydrate was added in the suspension with continuous stirring followed by mixing saccharine sodium (sweetener) and peppermint. The stirring process of the total polymeric suspension was continued for 6 hrs, and 25 mL of the solution was casted in polypropylene petri plates (Polylab Industries Pvt., Ltd., India) and kept overnight to remove the entrapped air bubbles. The suspension was dried at 45°C, and wafers were cut with in-house fabricated hollow punch (dia. 2.2 cm) and kept in desiccators, maintained relative humidity (60 ± 5%) until further analysis [12]. The thickness of each wafer was measured using a micrometer (Mitutoyo, Tokyo, Japan) at five locations (centre and four corners), and the mean thickness was calculated. Samples with air bubble, nicks, or tears were excluded from analysis. All the 9 experimental batches were designated as FNA with numerical suffix from 1 to 9 in accordance with the experimental design elaborated in Tables 1 and 3. The composition of the wafers casting solution for formulation coded as FNA 1 was detailed in Table 2.

### 2.4. Evaluation of Buccoadhesive Pharmaceutical Wafers

#### 2.4.1. Swelling Index Study [14, 15]

Three wafers (surface area: 3.80 cm^2^) were tested for each formulation. Initial diameter of the wafers was recorded and kept them on the surface of an agar plate (2% m/v) maintained at 37°C. Measurement of the diameter of the swollen patch was done at 1 h. Radial swelling was calculated from the following equation:

(1) $$\begin{array}{c} S_{D}\left(\%\right)=\frac{D_{t}-D_{o}}{D_{o}}\times 100, \end{array}$$

where *S*_*D*_ (%) is the percent swelling obtained by the diameter method, *D*_*t*_ is the diameter of the swollen wafer after time *t*, and *D*_*o*_ is the original wafer diameter at time zero.

#### 2.4.2. Disintegration Study [16]

**Figure 1:**
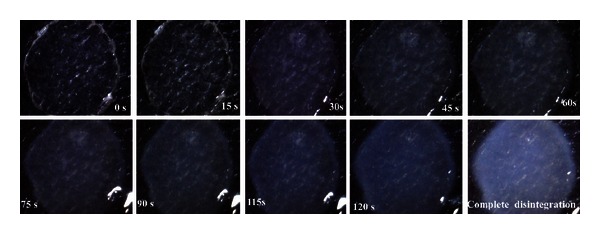
Scanning electronic microscopic photographs (250X) of the wafer disintegration process at different time intervals.

The wafer size required for dose delivery (3.80 cm^2^) was placed on a glass petri dish containing 10 mL of distilled water. The time required for the wafer to break was noted as *in vitro* disintegration time. All experimentation was done in triplicate. The disintegration process was visualized with optical scanning electronic microscope (RLMSCOPE, model number SM1500, Bikash Scientific Instruments, WB, India). The photographs were given in Figure 1.

#### 2.4.3. Surface pH Measurement

Surface pH of the wafers was measured according to the method described elsewhere [14, 15]. The wafers were left to swell for 10 min on the surface of an agar plate. The surface pH was measured by means of a pH paper placed on the surface of the swollen wafer. The mean of three readings was recorded.

### 2.5. Investigation of Drug-Excipient Interactions

#### 2.5.1. ATR-FTIR Spectroscopy of Prepared Wafers

**Figure 2:**
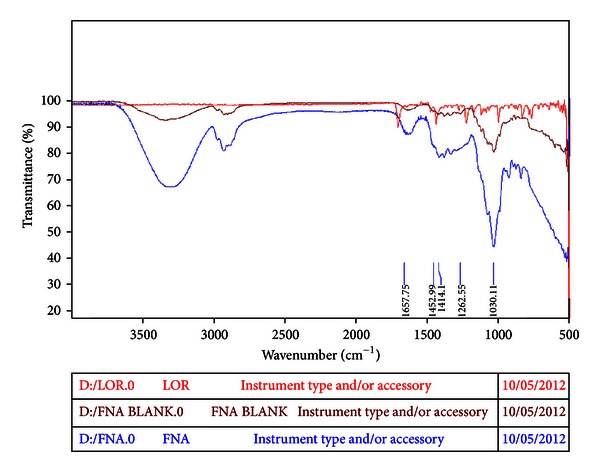
FTIR spectrum of LOR, blank wafer, and drug-loaded wafer formulation (FNA 3).

 Attenuated total reflectance Fourier transformed infrared spectroscopy (ATR-FTIR) of blank wafer, LOR, and drug-loaded wafer formulations (FNA) was recorded on a Bruker-ALPHA FTIR spectrophotometer with Opus 6 software. The ATR-FTIR spectra of LOR, blank wafer, and drug-loaded wafer formulation (FNA 3) were given in Figure 2.

#### 2.5.2. Differential Scanning Calorimetry

**Figure 3:**
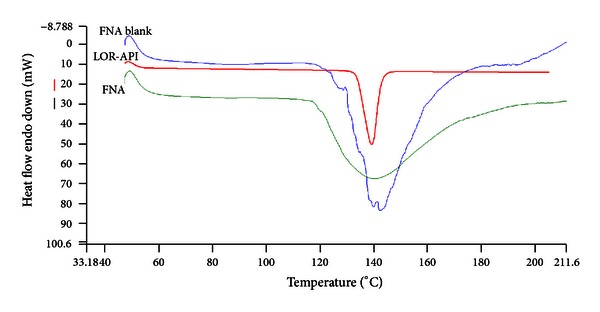
DSC thermograms of LOR, blank wafer (FNA blank), and drug-loaded wafer formulation (FNA 3).

Thermal properties of blank wafer (without drug), LOR, and drug-loaded wafer (FNA 3) were characterized using thermal analyser (Perkin Elmer, USA, Model—JADE DSC). Nitrogen at the rate of 20 mL/min was used as purge gas and at a varied temperature range of 26°C to 250°C at heating rate of 10°C/min under a nitrogen atmosphere. Calibration for the temperature and heat flow was carried out using a pure alumina as internal standard at the same heating rate of the experiments. The DSC thermograms of LOR, blank wafer, and drug-loaded wafer formulation (FNA 3) were shown in Figure 3.

#### 2.5.3. X-Ray Diffraction (XRD) Study of Prepared Wafers

**Figure 4:**
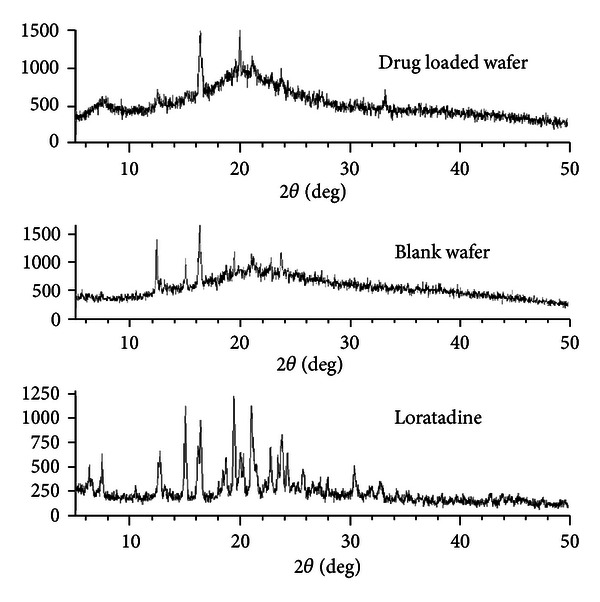
X-Ray powder diffraction pattern of Loratadine, blank wafer, and drug-loaded wafer formulation (FNA 6).

 Powder XRD pattern of LOR, blank wafer, and drug-loaded wafers was recorded to gain information about the state of drug in the wafers using diffractometric system (Rigaku Make Ultima-III, Japan) at 1.5 mA and 30 KV over the range 2*θ* = 5° to 50° at rate of 2*θ* = 5°/min. The X-ray diffractogram of LOR, blank wafer, and drug-loaded formulation (FNA 6) was shown in Figure 4.

### 2.6. *In Vitro* Measurement of Buccoadhesion [17, 18]

**Figure 5:**
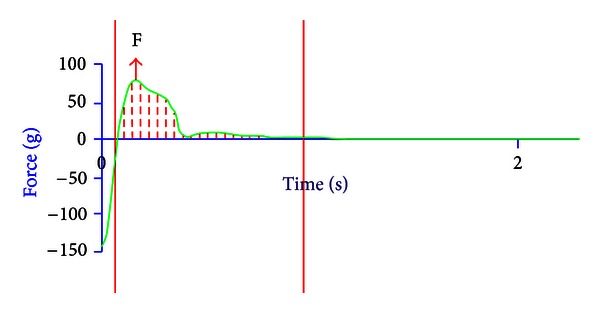
Representative graph of the *in vitro *bioadhesion test (FNA 6).

The *in vitro *bioadhesion properties of the pharmaceutical wafers were assessed on bovine buccal mucosa as a model membrane, using a TAXT2i texture analyzer. The buccal mucosal tissue was obtained from a local slaughter house, cleaned, washed, and stored at −20°C. Preserved buccal mucosa was hydrated with simulated saliva solution and allowed to reach normal room temperature (25°C) before commencement of the experiment, and it was tied to the lower probe of the assembly. The wafer was attached to the upper probe of the assembly using double-sided adhesive. The upper probe moves towards the lower probe with test speed 0.5 mm/sec and posttest speed 1 mm/sec. The wafer was allowed to adhere to the bovine buccal mucosa membrane with applied force 150 g, return distance 10 mm. The experiment was carried out at room temperature (25°C). The resultant force time plot of a pharmaceutical wafer formulation (FNA 6) was shown in Figure 5.

### 2.7. *In Vitro* Release Study [12, 16, 19]

The *in vitro* drug release of the wafers was determined using a paddle type dissolution apparatus (Excel Enterprises, Kolkata, India). In order to mimic the *in vivo* adhesion and to prevent the wafers from floating, each wafer was fixed to a rectangular glass slab and placed at the bottom of the dissolution vessel prior to starting the dissolution test. The dissolution medium was 250 mL of simulated salivary fluid. The rotation speed was 50 rpm at 37°C ± 0.5°C. The drug release was analysed spectrophotometrically at 248 nm. Every 30 s, 5 mL samples were manually withdrawn, filtered through a 0.45-*μ*m membrane filter, and analysed by UV-VIS spectroscopy (Thermo Scientific UV1). The withdrawn amount of dissolution medium was calculated. The measurement was made in triplicate with the standard deviation as a measure of variation.

### 2.8. Optimization Data Analysis [3, 20]

Various RSM computations for the current optimization study were performed employing Design-Expert software (Trial version 8.0.7.1, Stat-Ease Inc., Minneapolis, MN, USA). Statistical second-order model including interaction and polynomial terms was generated for all the response variables. The general form of the model is represented as in the following:

(2) Y=β0+β1A+β2B+β3AB+β4A2+β5B2+β6A2B+β7AB2+β8A2B2,

where *β*_0_, the intercept, is the arithmetic average of all quantitative outcomes of nine runs, *β*_1_ to *β*_8_ are the coefficient computed from the observed experimental values of *Y*, and *A* and *B* are the coded levels of the independent variable(s). The terms *AB* and *A* ^2^ and *B* ^2^ are the interaction and polynomial terms, respectively. The main effects (*A* and *B*) postulate the average result of changing one factor at a time from its low to high value. The interaction term (*AB*) shows how the response changes when two factors are changed accordingly. The polynomial terms (*A* ^2^ and *B* ^2^) symbolize nonlinearity. The polynomial equation was used to draw conclusion after considering the intensity of coefficient and the mathematical sign it carries, that is, positive or negative. A positive sign signifies synergesis. Statistical validity of the polynomials was established on the basis of ANOVA provided in the Design-Expert software. Level of significance was considered at *P* < 0.05. Also, three-dimensional response surface graphs and contour plots were generated by the Stat-Ease Design-Expert software.

## 3. Result and Discussion

### 3.1. Physicochemical Analysis

The physicochemical parameters of the prepared wafers are summarized in Tables 3 and 4. The thickness of the wafers varied within the range from 200 *μ*m to 556 *μ*m. The pH of the wafers was within the acceptable range and exhibited sufficient mechanical strength rendering it nonirritant to the mucosal surface and ensuring flexibility of the wafers, respectively. These properties warrant the ease of use of the product by the patient thereby endorsing the appropriate selection of the plasticizers.

### 3.2. Drug-Excipient Interactions

 ATR-FTIR spectra of LOR revealed major peaks at 1700.66 cm^−1^, 996.17 cm^−1^, 1433.63 cm^−1^, and 1220.46 cm^−1^. An absorption around 1700 cm^−1^ was attributed to amide group due to C=O stretching and N–H deformation. Absorption at 996 cm^−1^ was attributed to aryl halide group due to C–Cl stretching. Absorption at 1433 cm^−1^ was assigned to nitro compound due to N=O stretching. Absorption at 1220 cm^−1^ was observed due to saturated aliphatic and side chain aromatic groups present in the structure of LOR [21]. The ATR-FTIR study reveals that the absorption peaks of formulation codes as FNA 3 are within the range as shown in LOR absorption peaks. No drastic change attributed to drug-excipients interaction and/or incompatibility was observed in the ATR-FTIR spectra shown in Figure 2.

### 3.3. DSC Analysis

This was again reasserted with the DSC study of the pure drug Loratadine, blank wafer, and drug-loaded wafer (Figure 3). For pure LOR, a sharp endotherm was recorded at 139.31°C corresponding to the melting point of the pure drug. The thermal behaviour of the prepared drug-loaded wafer formulation (FNA 3) showed endotherm at 138.87°C. The depression of the endothermic peak in drug-loaded wafer suggested that LOR could be present in an amorphous form in the matrix of the wafer, and its behaviour would be expected to amend the solubility of the drug in water, resulting in better bioavailability.

### 3.4. XRD Analysis

 Molecular structure analysis of the prepared wafers through X-ray diffraction study confirmed the amorphous state or molecular dispersion of LOR in the prepared wafers. This was further supported by the absence of prominent signals (Figure 4) of LOR in the prepared wafers (FNA 6). Though some signals were obtained in the blank and prepared wafers, that could be the characteristic signal for lactose monohydrate, which may be due to its molecular rearrangement during wafer preparation [22] or due to propylene glycol attributed partial solubilisation that converts LOR to a molecular dispersion in the wafer matrix, thus leading to low intensity signal as observed in the wafer in comparison to pure drug. 

### 3.5. Effect of Formulation Variable on Buccoadhesion Force (*Y*_1_)

Table 3 listed the values of various response parameters of the nine optimization batches. The constant and regression coefficients for *Y*_1_ (bioadhesion force) were as follows:

(3) Y1=31.04+8.60∗A+1.93∗B+17.03∗A∗B+23.33∗A2−1.37∗B2.

The polynomial quadric model was found significant with an *F* value of 11.26 (*P* = 0.0369). 

**Figure 6:**
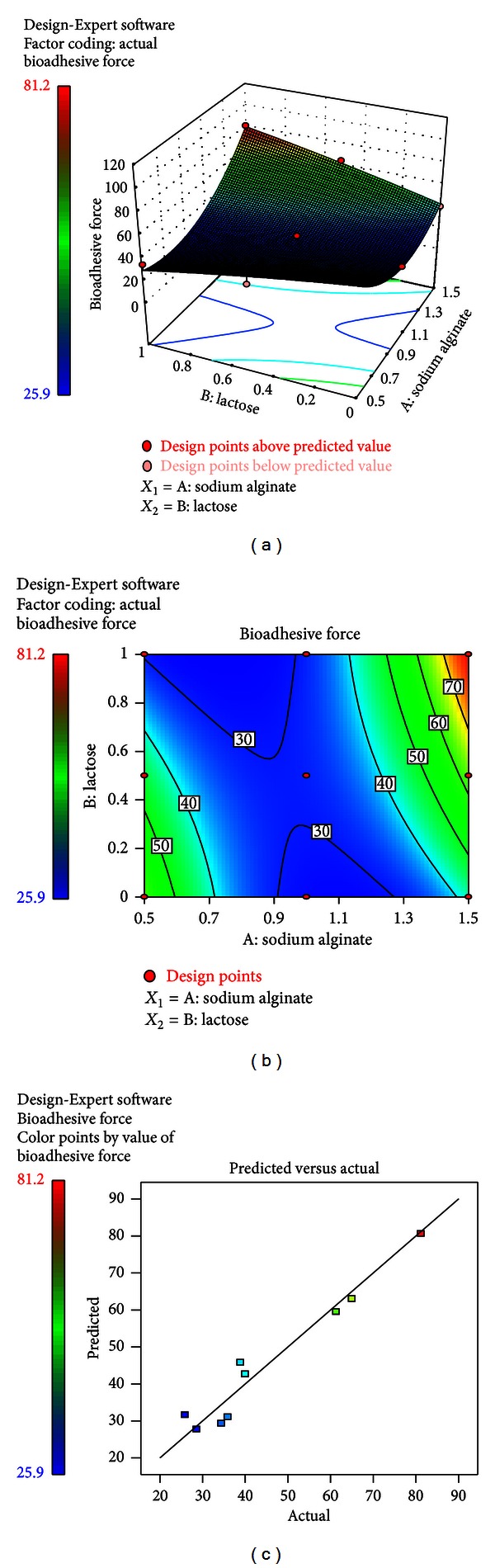
(a) Response surface plots showing the influence of sodium alginate and lactose on the bioadhesive force (*Y*_1_). (b) Corresponding contour plot showing the relationship between various levels of the two factors. (c) Plot between observed and predicted values of *Y*_1_.

The value of correlation coefficient (*r* ^2^) was found to be 0.9494. Equation (3) indicated that both *AB*, *A* ^2^ were significant model terms. The combination effect of factors *A* and *B* could further be elucidated with the help of response surface and contour plots (Figures 6(a) and 6(b)). However, the steeper ascent in the response surface with sodium alginate (*A*) than with lactose monohydrate (*B*) was clearly perceptible from both the plots, indicating that the effect of sodium alginate was comparatively more pronounced than that of lactose monohydrate. From this discussion, conclusion can be drawn that the bioadhesion might be changed by appropriate selection of the levels of *A* and *B*, whereas Figure 6(c) represented the observed response values compared with that of the predicted values depicting a good fit. 

### 3.6. Effect of Formulation Variable on Disintegration Time (*Y*_2_)

When the model terms for *Y*_2_ (disintegration time of wafers) were fitted in the polynomial quadric model, they were found to be significant with an *F* value 58.84 (*P* = 0.0034). The quadratic model describing the disintegration time of wafers could be written as

(4) Y2=1.20+0.65∗A+0.17∗B+0.13∗A∗B−0.13∗A2−0.025∗B2,R2=0.9899.

**Figure 7:**
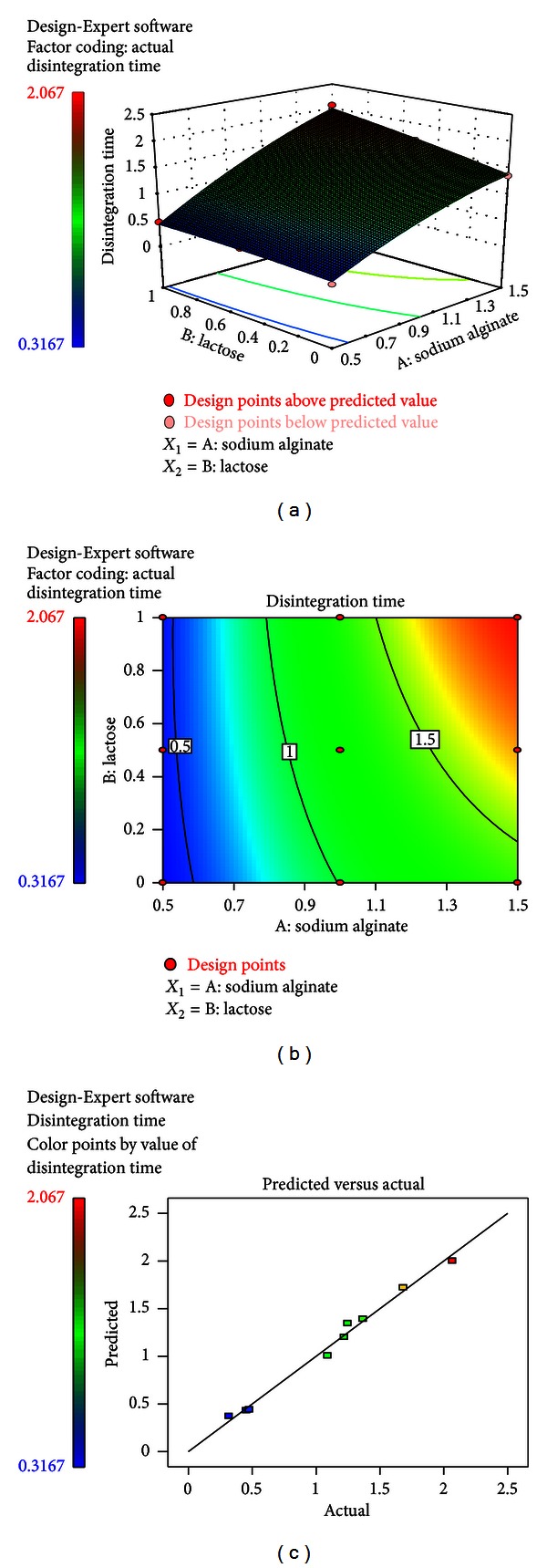
(a) Response surface plots showing the influence of sodium alginate and lactose on the disintegration time (*Y*_2_). (b) Corresponding contour plot showing the relationship between various levels of the two factors. (c) Plot between observed and predicted values of *Y*_2_.

In this case, effect of *A* and *B* was significant. The combined effect of factors *A* and *B* could further be elucidated with the help of response surface and contour plots (Figures 7(a) and 7(b)). A linear relationship was observed between response *Y*_2_ and factor *A* and *B*. The disintegration time of the wafer increased proportionately with the fraction of sodium alginate and lactose monohydrate.  Figure 7(c) represented the observed response values compared with that of the predicted values depicting a good fit.

### 3.7. Effect of Formulation Variable on Percent Swelling of the Wafers (*Y*_3_)

Swelling is an important parameter to be studied before considering bioadhesion [23]. Fitting the model term *Y*_3_ into polynomial quadric model, they were found to be significant with an *F* value 58626.95 (*P* ≤ 0.0001). The quadratic model describing the percent swelling index of wafers could be written as

(5) Y3=59.62+16.86∗A+0.24∗B−0.063∗A∗B+6.86∗A2+0.25∗B2,R2=1.0000.

**Figure 8:**
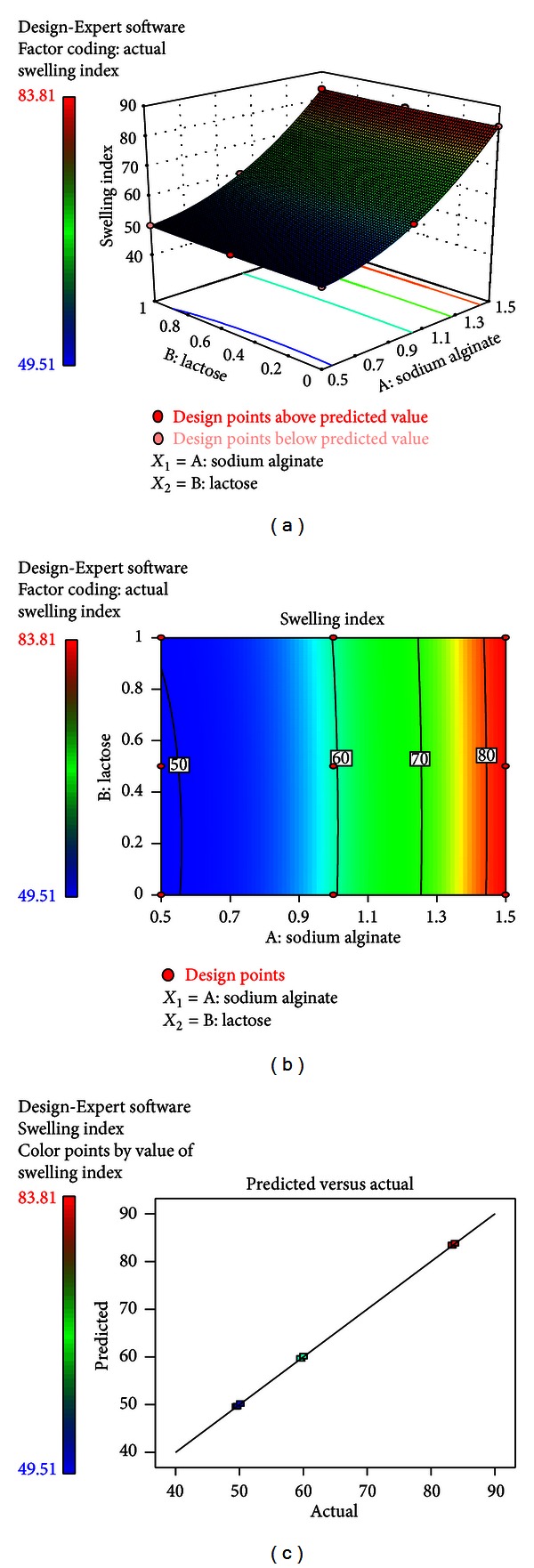
(a) Response surface plots showing the influence of sodium alginate and lactose on the percent swelling index (*Y*_3_). (b) Corresponding contour plot showing the relationship between various levels of the two factors. (c) Plot between observed and predicted values of *Y*_3_.

In this case, *A*, *B*, *A* ^2^, and *B* ^2^ were significant. The combined effect of factors *A* and *B* could further be elucidated with the help of response surface and contour plots (Figures 8(a) and 8(b)).  Figure 8(c) represented the observed response values compared with that of the predicted values depicting a good fit. A predominant effect of factor *A* was visualized from the equation that with increase in concentration of buccoadhesive polymer (*A*), the swelling property of the wafer also increases.

### 3.8. Effect of Formulation Variable on *t*_70%_ of the Wafers (*Y*_4_)

*t*_70%_ is an important variable for assessing drug release profile from the dosage form, indicating the amount of drug available at the site of absorption. This parameter was dependent on the formulation variables. The quadratic model for *t*_70%_ (*Y*_4_) was found to be significant (*P* = 0.0231) with an *F* value of 15.72. In this case, factor *B* was found more significant. Consider the following:

(6) Y4(t70%)=113.33+20.00∗A+70.0∗B−7.50∗A∗B−20.00∗A2+40.00∗B2.

**Figure 9:**
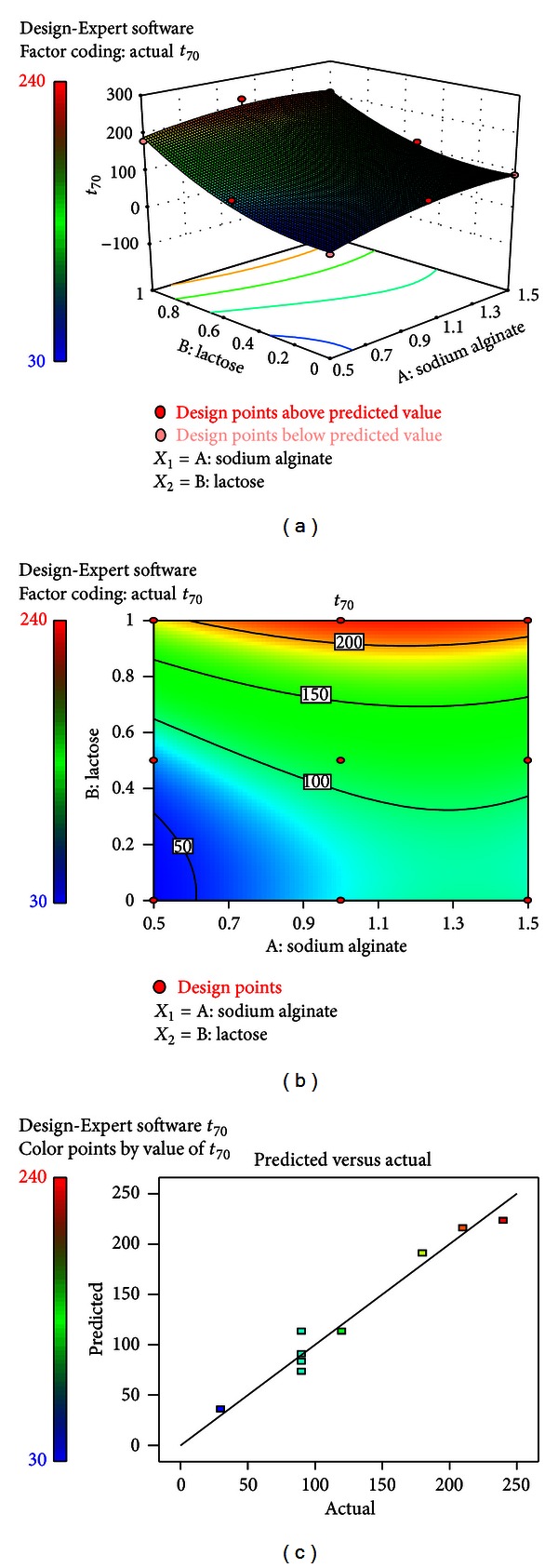
(a) Response surface plot showing the influence of sodium alginate and lactose on the *t*_70%_ (*Y*_4_). (b) Corresponding contour plot showing the relationship between various levels of the two factors. (c) Plot between observed and predicted values of *Y*_4_.

The combined effect of factors *A* and *B* could be explicated with the help of response surface and contour plots (Figures 9(a) and 9(b)).  Figure 9(c) represented the observed response values in comparison with the predicted values depicting a good fit. A clear effect was observed with increase fraction of lactose monohydrate at all three levels of sodium alginate. An increase in the amount of lactose monohydrate resulted decease in drug release. This might be due to the tight binding exhibited by lactose monohydrate in forming the hydrophilic matrix. Another reason of retarding the drug release might be attributed by a protective gel layer, caused by excess sodium alginate content that could be formed before water's entry in the matrix thus hydrating the inner layer.

The results of ANOVA for the dependent variables (Table 5) demonstrated that the model was significant for all response variables.

### 3.9. Optimization

**Figure 10:**
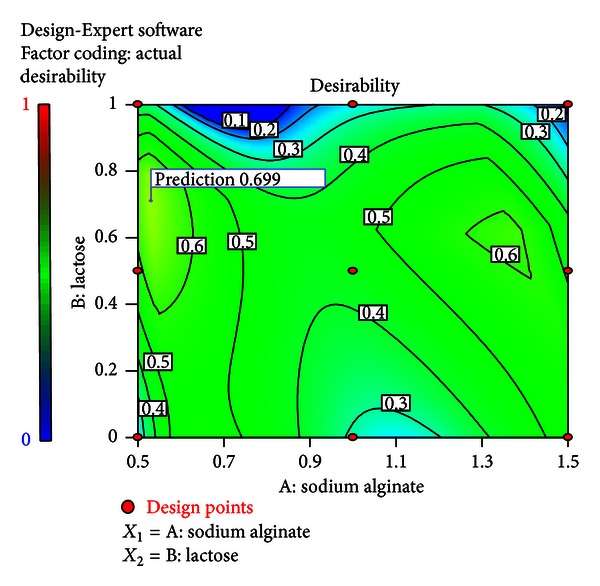
Contour plot showing the optimization procedure depending on numerical method.

An optimum setting for the formulation was generated by the numerical optimization technique following desirability approach. The process was optimized for the dependent (response) variables *Y*_1_ to *Y*_4_, and the optimized formula was reached by keeping the bioadhesion force in target of 53.33 gm. The disintegration time of the wafer was kept at 0.5 min (30 sec), percentage swelling index was kept in range of 49.51 and 83.81, and *t*_70%_ was kept at 135 sec. The formulation FNA 8 fulfilled nearly all the criteria set from the desirability search (Figure 10). The low % prediction error of −0.020 to 2.71 indicated the high prognostic ability of RSM [24] (Table 6).

## 4. Conclusion

In conclusion, we reported here the formulation of LOR-containing buccoadhesive wafers produced following design of experiment and optimized with the help of response surface methodology involving the factors as percentage of buccoadhesive polymer and hydrophilic matrix forming lactose monohydrate and responses taken as bioadhesive force, disintegration time, swelling percentage, and release (*t*_70%_) of drug from the wafers. The wafer formulation coded as FNA 8 was found to be optimized with desirable bioadhesive strength, disintegration time, swelling property, and optimum drug release in the buccal environment. One of the advantages of oral wafer preparation was the ease in intake. The present wafer base preparation was found to be easily dissolving in saliva without producing insoluble materials. The constituents of the wafer base preparation had already been used in internal dosage additives and thus safe. These findings when taken together suggest that the present formulated buccoadhesive wafers containing Loratadine can be reproduced with high predictability and shall be potentially useful to patients with dysphagia or aphasia that require an immediate relief from the allergic conditions.

## Figures and Tables

**Table 1:** Factor combination as per 3^2^ factorial design.

Trial number	Coded factor levels		
	Factor 1		Factor 2
1	1.000		−1.000
2	−1.000		−1.000
3	0.000		0.000
4	1.000		0.000
5	0.000		−1.000
6	−1.000		1.000
7	1.000		1.000
8	−1.000		0.000
9	0.000		1.000

Translation of coded levels in actual units			

Coded levels	−1.000	0.000	1.000
*X*_1_: Sodium alginate (% w/v)	0.5	1	1.5
*X*_2_: Lactose monohydrate (% w/v)	0	0.5	1

**Table 2:** Composition of the casting solution as per experimental design to prepare wafer formulation FNA 1 (trial number 5).

Ingredients	Code: FNA 1
Na Alginate	1% w/v
HPC	2% w/v
PG	1.5% w/v
Glycerin	1.6% w/v
Saccharine Na	0.1%w/v
Ethanol	15% v/v
Sorbitol	0.5% w/v
Lactose monohydrate	—
Peppermint	0.01% w/v
Loratadine	0.134% w/v
Water (q.s)	100

**Table 3:** Response parameter for Loratadine-loaded buccoadhesive pharmaceutical wafers prepared as per 3^2^ factorial design.

Formulation code	Formulation composition		Bioadhesive force	Disintegration time	% Swelling	*T*_70%_ (*Y*_4_)
	Sodium alginate (% w/v)	Lactose monohydrate (% w/v)	(*Y*_1_) (gm)	(*Y*_2_) (min)	index (*Y*_3_)	(sec)
FNA 1 (0, −1)	1.00	0.00	28.6	1.09	59.71	90
FNA 2 (0,0)	1.00	0.50	35.9	1.22	59.58	90
FNA 3 (0, +1)	1.00	1.00	25.9	1.247	60.08	240
FNA 4 (+1, −1)	1.50	0.00	40	1.367	83.39	90
FNA 5 (+1,0)	1.50	0.50	65	1.683	83.32	120
FNA 6 (+1, +1)	1.50	1.00	81.2	2.067	83.81	210
FNA 7 (−1, −1)	0.50	0.00	61.3	0.316	49.51	30
FNA 8 (−1,0)	0.50	0.50	38.9	0.453	49.68	90
FNA 9 (−1, +1)	0.50	1.00	34.4	0.476	50.18	180

**Table 4:** Characteristics of Loratadine-loaded buccoadhesive pharmaceutical wafers.

Formulation code	Thickness	Folding endurance	Surface pH
FNA 1	0.332 ± 0.011	≥50	6.7 ± 0.01
FNA 2	0.324 ± 0.024	≥50	6.7 ± 0.02
FNA 3	0.352 ± 0.020	≥45	6.8 ± 0.01
FNA 4	0.322 ± 0.029	≥55	6.85 ± 0.01
FNA 5	0.338 ± 0.033	≥50	6.86 ± 0.01
FNA 6	0.556 ± 0.021	≥40	6.8 ± 0.02
FNA 7	0.200 ± 0.017	≥45	6.8 ± 0.01
FNA 8	0.280 ± 0.014	≥50	6.8 ± 0.01
FNA 9	0.352 ± 0.027	≥40	6.7 ± 0.01

**Table 5:** Results of analysis of variance (ANOVA) for measured responses.

Parameter	SS	df	MS	*F*	Significance *F*
Bioadhesion (*Y*_1_)					

Model	2718.21	5	543.64	11.26	0.0369
Residual	144.80	3	48.27	—	—
Cor total	**2863.01**	**8**	—	—	—

Disintegration time (*Y*_2_)					

Model	2.77	5	0.55	58.82	0.0034
Residual	0.028	3	9.432*E* − 003	—	—
Cor total	**2.80**	**8**	—	—	—

% Swelling index (*Y*_3_)					

Model	1799.79	5	359.96	58626.95	<0.0001
Residual	0.018	3	6.140*E* − 003	—	—
Cor total	**1799.81**	**8**	—	—	—

*t*_70%_ (*Y*_4_)					

Model	36025.00	5	7205.00	15.72	0.0231
Residual	1375	3	458.33		
Cor total	**37400**	**8**	—	—	—

df: degree of freedom; SS: sum of square; MS: mean square; *F*: Fischer's ratio.

**Table 6:** Predicted and observed response variables of the optimal buccoadhesive wafer of Loratadine.

	*Y*_1_	*Y*_2_	*Y*_3_	*Y*_4_
Predicted	39.434 ± 6.947	0.4419 ± 0.0971	49.786 ± 0.078	111.326 ± 21.408
Observed	38.9	0.453	49.68	90
Predicted error (%)	−1.354	2.71	−0.200	0.100
